# Is There Any MRI Pattern That Discriminates Female From Male Migraine Patients?

**DOI:** 10.3389/fneur.2019.00961

**Published:** 2019-09-06

**Authors:** Nasim Maleki, Xiao Michelle Androulakis

**Affiliations:** ^1^Psychiatric Neuroimaging Division, Department of Psychiatry, Massachusetts General Hospital, Harvard Medical School, Boston, MA, United States; ^2^Columbia VA Health Care System, Columbia, SC, United States; ^3^Department of Neurology, School of Public Health, University of South Carolina, Columbia, SC, United States

**Keywords:** brain, neuroimaging, magnetic resonance imaging, sex, migraine, sex-related differences

## Abstract

There has been accumulating evidence on sex disparity in incidence, prevalence, symptomology, and burden of migraine. Several neuroimaging studies on migraine patients attempted to unravel the mechanisms of the disease, yet very few of them examined the sex-related differences. Here, we will first discuss some of the reported neuroimaging patterns that discriminate females from males in migraine. We will then re-examine the salient neuroimaging findings in migraine and discuss them in relation to sex-related influences. Finally, we will discuss some of the intriguing recent data suggesting the presence of sex-specific traits in migraineurs. These findings may have potential implications for future neuroimaging studies to identify underlying correlating patterns in the brain to (1) explain the neural basis for higher prevalence of migraine in women, and (2) better understand migraine-specific changes during different stages of life in both men and women.

## Introduction

Despite advances in understanding of the migraine pathophysiology (1), as one of the most prevalent disabling disorders worldwide, migraine disease continues to be an unresolved major public health problem for both men and women (2–6). Of the 38 million migraine sufferers in the US, two-thirds are estimated to be female (7, 8) with differences in the incidence pattern appearing around puberty (9). Sex differences in migraine also extend to greater symptomology, higher rate of visual auras, higher headache-related disability, and greater healthcare resource utilization by females (10). In the past two decades, several neuroimaging studies have attempted to identify potential differences in the brains of migraineurs, however only a very limited number of studies have examined the sex-specific differences in the brains of migraineurs. In this review: (i) We will first discuss neuroimaging findings on the patterns that discriminate women from men in migraine to date; (ii) We will then re-examine some of the salient neuroimaging findings in migraine and discuss them in relation to the sex-related influences; (iii) Finally, we will discuss some of the intriguing recent findings that seem to suggest presence of sex-specific traits in migraineurs, which may have potential implications for future neuroimaging studies. These together may not only hold clues to the sex disparity in migraine, but also consequently shed more light on the mechanisms of the disease.

## Neuroimaging Findings on Sex-Related Brain Differences in Migraine

There are very limited neuroimaging studies with considerably small sample sizes that have examined sex-related differences in migraine. In a study on episodic migraineurs and matched healthy control individuals, increased cortical thickness in the insula and precuneus in female migraineurs and a smaller volume of the parahippocampal gyrus in male migraineurs were observed despite both male and female migraineurs having comparable disease frequency and duration (11). Functionally, women with migraine showed stronger response to pain in brain regions involved in emotional processing such as the amygdala, which was consistent with increased measures of pain related unpleasantness for them compared to men with migraine. In a follow up study, abnormality in the insula was again observed in women between the ages of 20–65 years with migraine. It was found that there was a lack of age-related thinning in the insular cortex in female migraineurs compared to female healthy controls (12). A meta-analysis of nine voxel-based morphometry neuroimaging studies (222 migraineurs and 230 healthy controls), suggested sex-influence on some of the observed differences in the gray matter volume between migraineurs and healthy subjects. The analysis showed that a higher percentage of females in the patient sample was associated with decreased gray matter in the right dorsolateral prefrontal cortex (13).

Sex-related differences in the topological properties of the brain functional networks have also been reported recently. In one study, a noxious stimulation paradigm utilizing a thermal probe was applied to the back of the hand in order to evoke a painful response (11). Female migraineurs showed greater brain activation in response compared to men with migraine in certain brain regions such as the amygdala, parahippocampus, basal ganglia, and posterior cingulate cortex. These regions are involved in processing of the emotional aspects of pain. The same study indicated significant differences between the functional connectivity of these structures with the rest of the brain (using a seed-based functional connectivity analysis approach), specifically with the areas involved in pain processing. Using graph theory analysis, one study revealed network level differences that may reflect faulty communication within and between brain regions in female migraineurs (14). Another study has further revealed widespread disrupted functional connectivity in female migraineurs compared to healthy women primarily in brain regions involved in discriminating sensory features of pain, pain modulation, and sensory integration (15). Sex-related differences have also been reported in the incidence of white matter abnormalities in female migraine patients compared to age-matched healthy female controls with no such difference in males (16).

## Neuroimaging Findings in Migraine and Potential Sex-Related Influences

### Hypothalamic Involvement

One of the most consistent and salient findings in neuroimaging studies of migraine is abnormal hypothalamic activity preceding (17, 18), during (18–20) and even in between the migraine attacks (21). Most of the premonitory autonomic symptoms associated with a migraine attack are indeed thought to be of hypothalamic origin (22, 23). The hypothalamic orexinergic system in particular is thought to be a key regulator of the modulatory effects of the hypothalamus on the trigeminovascular system implicated in migraine pathophysiology (24). Orexin, a neuropeptide solely synthesized in the hypothalamus, plays a major role in modulating brain activity and a variety of complex functions including sleep, reward, feeding behavior, and stress response (24). Functional changes in hypothalamo–brainstem connectivity (22) including changes in functional coupling with the spinal trigeminal nuclei and the dorsal rostral pons (25) are shown to precede a migraine attack. The hypothalamus also serves as an interface between the neural system and the peripheral endocrine systems. It is likely that cyclic activation of trigeminovascular system by sex hormones during menstrual cycles may be one of the contributing factors to the incidence of migraine attacks via coupling with the hypothalamus in women. However, to the best of our knowledge there have not been any reports on neuroimaging differences between male vs. female migraineurs involving the hypothalamus.

### Insular Involvement

Insular abnormalities in association with migraine have been reported in several neuroimaging studies (26–31). There is abnormal intrinsic connectivity between the anterior insula and primary sensory cortices, and the pons (32). There is abnormal connectivity of the default mode network and central executive network in migraineurs compared to healthy subjects (29). Chronic migraine disease duration is correlated with intrinsic functional connectivity strength between the anterior insula and mediodorsal thalamus and the anterior insula and periaqueductal gray. Higher frequency of migraine attacks mediates increased connectivity between the somatosensory cortex and the anterior insula in response to evoked pain (31). Aberrant functional connectivity between right orbitofrontal insula and prefrontal regions is also observed within the salience network in women with chronic migraine (33). The insula is one of the regions that has been implicated in neuroimaging studies of sex-related differences in migraine.

### Brainstem Involvement

Multiple studies have reported abnormal brainstem function in ictal and interictal migraineurs. This includes increased neuronal activity in the brainstem during migraine attacks (26, 34–36) and dysfunctional descending modulation, involving the periaqueductal gray (PAG) and dorsal rostral pons (36), during and between migraine attacks (37–39). Moreover, during the pre-headache phase of a migraine attack (<24 h), increased infra-slow oscillation and homogeneity in dorsal pons, spinal trigeminal nucleus, and hypothalamus are observed in migraine patients (40). Interictally, the dorsal pons show increased connectivity with the bilateral anterior insula in migraineurs (32). In an animal study, CGRP expression increased within the PAG in ovariectomized female rats, and CGRP level remained elevated even after receiving hormone replacement therapy (41). To the best of our knowledge, no studies evaluated male vs. female brainstem functional/structural differences in migraine. Given that a lack of female sex hormone increased CGRP expression in PAG in ovariectomized female rats (41), it is likely that descending pain modulation is affected differently in opposite sexes. Therefore, investigation of neuroimaging patterns in migraine should shed light on how sex influences pain modulation.

### Extended Amygdala Involvement

The extended amygdala, which consists of the central medial amygdala, sublenticular substantia innominata, the nucleus accumbens shell, and the bed nucleus of the stria terminalis, regulates nociception, aversive motivational state, reward, memory, and learning (42). It interconnects extensively with the thalamus, hypothalamus, and cortical regions (43) and as such plays an important role in neural circuitry of emotion regulation (44). In a resting state fMRI study that investigated salience network connectivity in women with chronic migraine, the bilateral central and medial amygdala were found to be significantly less connected functionally with each other, and the overall salient network circuitry dys-synchronization was found to be centered on the extended amygdala among 351 salient intranetwork connectivities investigated (33). Using PET scan with u-opioid receptor tracer, researchers found that right amygdala opioid dysfunction is largely explained by migraine frequency and severity (45). Cortical spreading depression, a pathophysiological substrate of migraine with aura, was elicited in rats with NMDA administrated to the amygdala (46). The amygdala also shows sex differences in animal and human studies (47). These sex-specific differences in regional anatomy may explain the inconsistent findings amongst studies when including mixed (male and female) cohorts.

It seems reasonable to postulate that the extended amygdala is crucial (but not sufficient by itself) for the lack of habituation to salient information seen in migraine. Therefore, it contributes to the maladaptive response to head pain and promotes pain catastrophizing and recurrent negative thoughts commonly seen in female migraine patients.

### Network Level Differences

Intrinsic functional brain networks (IFBN) such as the Default Mode Network, Salience Network, and Central Executive Network are brain state-dependent, spatial topographies representing inter-regional connectivity patterns, and consisting of functionally correlated brain regions. The diverse symptomatology of migraine suggests that multiple functional brain regions are at play. Interestingly, decades of migraine neuroimaging research failed to confirm a single brain region responsible for its pathogenesis. From an evolutionary standpoint, each human brain region has adapted to take on multiple roles in different contexts in order to perform a variety of functions (48). Taken together, it is unlikely that one isolated part of the nervous system is sufficient or necessary to orchestrate such complex brain process as migraine. The unique advantage of the functional brain network approach in studying migraine and chronic migraine is that it allows for a systemic and comprehensive approach to map out migraine symptoms to the underlying brain circuitry. This is a far better approach compared to the localization approach using a whole brain atlas, which likely results in missing the “forest” by only examining the “tree(s).”

Decreases in salience network and central executive network connectivity are correlated with chronic migraine headache frequency in women, suggesting that improving synchronization of these networks through therapeutic interventions may improve clinical symptoms and have potential to be used as a biomarker for monitoring disease progression and treatment response (49). The intrinsic functional connectivity between the brain networks can be modulated by the phase of the menstrual cycle and by the usage of oral contraceptive pills (50). Therefore, it is likely that the migraine burden or treatment response in women would be influenced by these factors.

## Sex Specific Traits in Migraineurs and Implications for Neuroimaging

### Perimenopausal Migraineurs

Perimenopause as a midlife transitional period in women is associated with significant changes in certain brain networks' underlying processes such as thermoregulation, circadian rhythms, sleep, and sensory processing (51). Fluctuations and decline in the levels of ovarian hormones during this period also have significant modulatory influences on brain function (52–55), which could have significant implications for neurological disorders, including migraine (56, 57). A recent study provides evidence for increased incidence of vasomotor symptoms in aging women with a history of migraine (58). This finding may be concordant with neuroimaging findings that have shown sex-specific and disease specific abnormalities in the structure and function of the insular cortex, the core cortical region for autonomic integration, in women with migraine. This further emphasizes a need for neuroimaging studies of migraine in the aging population (59).

### Trait Estradiol Decline

In migraine, decline in estrogen levels is thought to be one of the most potent triggers for occurrence of a migraine attack and is commonly referred to as the “estrogen withdrawal hypothesis” (60, 61). A recent study has shown that women with a history of migraine have faster decline of estradiol prior to menses than women with no history of migraine, irrespective of whether they had experienced a headache in that cycle or not (62), suggesting there exists an endogenous trait in women with migraine. Changes in estrogen levels could have modulatory effects on neurons containing estrogen receptors and may increase nociception (63, 64). Estrogen receptors are widely expressed in the trigeminal sensory system (65). The effects of estrogen on the receptors could be through modulating expression of nociceptive mediators, as well as through receptor coupling. Increased release of excitatory neurotransmitters can lead to the sensitization of the trigeminovascular system leading to peripheral and central sensitization (66, 67).

Neuroimaging studies in menstrual cycling women with migraine according to the phase of their menstrual cycle should provide insights on how decline in estrogen might modulate or affect functional activity or connectivity. Studying the interactions between sex hormones and brain activity should also extend to men as the dynamics of such interactions might not be the same in men and women. In fact, estrogen may also play a role in migraine for men but surprisingly men with migraine exhibit increased levels of estradiol while exhibiting clinical evidence of relative androgen deficiency (68).

### Pubertal Development and Onset of Migraines

The highest incidence of migraine coincides with pubertal development period, which is also a critical period for brain reorganization. The sex-specific differences in timing and speed of these changes may be critical in reorganization of connections in the brain, and therefore, may predispose individuals to various diseases, such as migraine, with sex disparity. Recent studies provide support for this notion by revealing that the “timing” of the onset of menarche matters in migraine: earlier age at menarche increases the risk of migraine, but not other types of headaches, in women by adulthood (69). It is likely that sex-specific differences in the brain of adults with migraine (11, 12) may have started to appear around the onset of puberty (70) and that the sex-specific traits in female migraineurs may have begun to crystalize during the same time. Sex-specific differences in the trajectory of development of brain regions that are implicated in migraine pathophysiology, such as brainstem nuclei, may also increase the susceptibility to migraine (71).

### Treatment Response Differences

FDA has recently approved several anti-calcitonin gene-related peptide (CGRP) therapies following successful randomized, placebo controlled, double blinded trials, all of which had predominantly female participants. None of these studies evaluated the treatment response differences in women vs. men. In animal models, CGRP triggers migraine-like response in female but not male rodents, suggesting that female-specific mechanisms may be involved consequent to CGRP receptor activation and that blocking CGRP is probably unlikely to work in males (72). There is also evidence for sex differences in the expression of CGRP receptor components in the spinal trigeminal nucleus with higher levels of expression in females (73). Therefore, we question if monoclonal antibody blocking CGRP may be as effective for migraine in men compared to women.

Very few studies have looked at the neuroimaging changes following migraine treatment. In a pilot longitudinal fMRI study, the impact of SPG treatment on salience and executive networks in women with chronic migraine was examined (74). It was found that total network synchronization improved in the executive network but not in the salient network. There was a trend toward improvement in the salient network but its insignificance was probably due to small sample size. Moreover, within the salience network, connectivity between prefrontal to limbic regions greatly improved. Comparing chronic migraine patients who responded to Botox vs. those who did not, responders showed improved functional connectivity in a small case control study (75).

## Conclusion

The sex-related differences in migraine go beyond the difference in prevalence of the disease and extend to sex-related differences in incidence and disease progression as well as in pharmacologic treatment response patterns. Given the sex differences in migraine, it may be more informative if researchers studied male and female migraineurs separately, such as using stratification in the study designs, to better delineate the underlying pathophysiology and treatment response. Statistical adjustment for sex differences in regression models does not and cannot adjust for the complexity of the underlying biological differences between males vs. females, and the sex-influences should be considered from the conception of neuroimaging studies to the analysis and interpretation of the results. This is certainly a dilemma when designing a migraine study given the already 1:3 male to female ratio in migraine and lesser engagement of men in research studies. At this point, it almost seems that we know less about male-specific compared to female-specific neuropathology. It is likely that despite the major overlap in the neural “culprits” involved, the modulatory influence of sex-influences will have a wider impact on the functional dynamics of the known players in migraine pathophysiology and as such findings in one sex may not simply and directly translate or extend to another sex.

## Author Contributions

All authors listed have made a substantial, direct, and intellectual contribution to the work, and approved it for publication.

### Conflict of Interest Statement

The authors declare that the research was conducted in the absence of any commercial or financial relationships that could be construed as a potential conflict of interest.
